# Condition Monitoring of Pneumatic Drive Systems Based on the AI Method Feed-Forward Backpropagation Neural Network

**DOI:** 10.3390/s24061783

**Published:** 2024-03-10

**Authors:** Monica Tiboni, Carlo Remino

**Affiliations:** Department of Mechanical and Industrial Engineering, University of Brescia, via Branze, 38, 25123 Brescia, Italy; carlo.remino@unibs.it

**Keywords:** diagnostics, classification, vibration signals, pneumatic actuators, spectral analysis

## Abstract

Machine condition monitoring is used in a variety of industries as a very efficient strategy for equipment maintenance. This paper presents a study on monitoring a pneumatic system using a feed-forward backpropagation neural network as a classifier and compares the results obtained with different sensor signals and associated extracted features as input for classification. The vibrations of the body of a pneumatic cylinder are acquired using both common industrial sensors and low-cost sensors integrated into an Arduino board. Pressure sensors for both chambers and a position sensor are also used. Power spectral density (PSD) is used to extract features from the acceleration signals, as well as statistical indices. Statistical indices are considered for pressure and position sensors. The results, which are based on experimental data obtained on a test bench, show that a feed-forward neural network makes it possible to identify the operating states with a good degree of reliability. Even with low-cost instrumentation, it is possible to realize reliable condition monitoring based on vibrations. This last result is particularly important as it can help to further increase the uptake of this maintenance approach in the industrial environment.

## 1. Introduction

Maintenance is crucial for manufacturers and operators, as the condition of a plant and machinery must be checked regularly to maintain their performance: only in this way production can meet expectations in terms of quality and economy, and failures during operation do not increase costs and reduce competitiveness. For this reason, diagnostic techniques are constantly evolving in terms of hardware (sensors, networks, processing units, data storage) and software (algorithms for data extraction, processing, and analysis) [1,2]. Since maintenance based on replacing the faulty component does not prevent failures in operation, which in the worst case can be harmful to the equipment or machine, more efficient and economical approaches have long been established [3]. Condition-based maintenance (CBM) is a maintenance program that collects process data in real-time (called condition monitoring—CM) to intercept phenomena that can lead to failures so that operators and manufacturers can make informed maintenance decisions to maintain machine availability, reliability, and performance. CBM can be applied to a variety of systems: general mechanical systems with shafts, gears, bearings [4,5,6,7,8,9,10,11], aircrafts [12], actuators [13,14], wind turbines [15,16,17] etc. CBM is generally based on diagnostics and/or prognostics: in the former, anomalies in process data caused by incipient failures are identified; in the latter, failures are predicted before they occur. In both cases, the goal is not only to minimize equipment failures and thus increase equipment availability but also to reduce planned maintenance and extend component life. Specifically, the diagnosis is based on: (i) knowledge of the observed process; (ii) diagnostic search strategy [18,19]. However, the research strategy depends heavily on what is known about the system (a priori knowledge), so diagnostic systems are divided into two broad categories based on (i) process knowledge (model-based knowledge) and (ii) process history knowledge [20]. Model-based solutions, in turn, are further divided into two categories: (i) quantitative [18]; (ii) qualitative [6,21]. Therefore, diagnostics do not necessarily require that the relationships between inputs and outputs are known, and a machine can be considered a black box. In this case, artificial neural networks (ANNs) can be useful because they can model highly nonlinear systems [22,23]. Several publications explored the ability of neural networks to detect and classify faults in pneumatic elements, especially valves. De Freitas et al. [24] used neural networks as models and classifiers: a first network was trained to predict the correct output of the valve, while a second network compared the energy of the signals provided by the model and the valve to detect any deviation that might reveal the presence of incipient faults. Karpenko and Sepehri [25] identified incorrect supply pressure, diaphragm leakage, and vent blockage faults by using nine features from the signature curves (valve position-pressure) and the dynamic error band (input signal-valve stroke) to train a multilayer feed-forward network. Subsequently, the authors [26] solved the same problem using performance features extracted from the valve response to a step command. Subbaraj and Kannapiran [27] developed a classifier capable of detecting nineteen faults, which was improved in later work by reducing the size of the inputs to the network through a principal component analysis (PCA) [28] and then compared to an adaptive neuro-fuzzy inference system (ANFIS) [29]. In the area of valve actuator systems, DAMADICS (Development and Application of Methods for Actuator Diagnosis in Industrial Control Systems) was developed, a benchmark for evaluating and comparing fault detection and isolation (FDI) [30]. The benchmark identifies nineteen faults divided into four categories: Control Valve Faults, Pneumatic Servo Motor Faults, Positioner Faults, and General Faults/External Faults. This benchmark was also used to evaluate the effectiveness of FDI systems based on neural networks. Kourd et al. [31] followed a similar approach to Gomes de Freitas et al. but errors were identified based on the Euclidean distance between the output of the valve and the output of the predictive model, one for each fault. Deng et al. [32] used the same method as Karpenko and Sepehri [25] by training the neural classifier with seven features obtained from the step response of the valve. Sundarmahesh and Kannapiran [33] improved the behavior of a classifier by reducing the input data through a principal component analysis (PCA). Prabakaran et al. [34] developed an initial system similar to that of Kourd et al. [31], and then developed a second system based on a self-organizing map that can adapt in real-time to the observed system [35]. Kowsalya and Kannapiran [36] confirmed that PCA improves the accuracy of a classifier. Andrade et al. [37] developed a system with neural networks as predictors and classifiers: a nonlinear autoregressive neural network model with exogenous inputs (NARX) provided the ideal system output, which was compared to the output of the real system—the resulting residuals were used to train a set of neural networks, one for each fault, which was then identified by a set of rules implemented in a decision tree. Demetgul et al. [38] were, to our knowledge, the only ones to use NNs to detect faults not in individual components but in an entire pneumatic system, in their case a Festo training system. The authors compared the results of two networks trained to detect eleven types of faults using eight signals from different sensors (pressure, potentiometer, switch). The two networks differed in the learning method: (i) unsupervised (adaptive resonance theory 2, ART2); (ii) supervised (backpropagation). Both networks provided good results.

Appropriate normal and fault training and test samples are necessary for intelligent fault diagnostic systems. A substantial amount of training samples is essential for the successful implementation of AI models. Class imbalance brought on by a lack of well-identified fault samples is one issue that might occur when using AI for anomaly detection. Several authors have suggested techniques to address imbalances in training samples, including closest neighbors interpolation, synthetic minority oversampling technique (SMOTE) [39], an approach that combines numerical simulation with Generative Adversarial Networks (GANs) [4], random oversampling, and others. The limitation of defective training samples may be inexpensively solved by numerical simulation utilizing the dynamic model of mechanical systems. Xiang et al. [5] provide the fundamental concept of a novel customized fault diagnostic approach that focuses on a shaft fault diagnosis. Three steps are involved in the process. The defective shaft’s fault-induced finite element method (FEM) model is first built. Additionally, the defective shaft’s vibration data are acquired by numerical modeling. WPT (wavelet packet transform) is used in the second stage to break down the vibration signal into its components. The training samples for the support vector machine (SVM) are produced by computing specific time-domain feature parameters for each of the signal components. Ultimately, the WPT-decomposed measured vibration signal and its constituent parts function as a test sample for the SVM that has been trained. Finally, the fault kinds are identified.

The research presented in this article investigates a neural-network-based approach for the condition monitoring of pneumatic drive systems. Vibration, acceleration, pressure, and position signals are monitored and used to extract features. One of the objectives of the analysis is to determine whether it is possible to achieve acceptable performance by adopting a low-cost vibration sensor and the corresponding signal acquisition solution. In addition, different inputs of the neural network are compared with each other and a sensitivity analysis of the network parameters is performed.

Vibrations are widely used to determine the state of a machine, not only because they react immediately when the state changes, but also because there are signal-processing techniques capable of extracting information even when it is weak and obscured by noise. They are therefore suitable for both continuous and intermittent monitoring. The pressures in a pneumatic system are easy to monitor and are related to possible air leaks.

The features used are the power spectral density (PSD), the fast Fourier transform (FFT), and the statistics of the vibration and pressure signals. For the statistical features, the aim was to identify a set of features for which the correlation with faults is robust enough to make them reliable features for machine health detection.

The novelty of the proposed work concerns the study of the possibility of using low-cost sensors for the detection of vibrations, whose signals can be acquired without expensive associated instruments. Moreover, there is no in-depth parametric sensitivity analysis of vibration-based condition monitoring with backpropagation neural networks applied to a pneumatic system in the literature.

The rest of the article is divided into the following sections: Section 2 presents the methodology and describes the experimental setups, the experiments, the analysis of the datasets, the extraction of the features, and the AI-based classifier used; the results of the classification are presented in Section 3 together with the sensitivity analysis performed; Section 4 is dedicated to the discussion of the results, comparing the different solutions analyzed, and the conclusions with the main findings are reported in Section 5.

## 2. Materials and Methods

This section is dedicated to the description of the methodology used, the test bench and the tests performed, the extraction of the features, a first analysis of the experimental signals obtained, and the structure of the selected neural network.

### 2.1. Methodology Description

**Figure 1:**
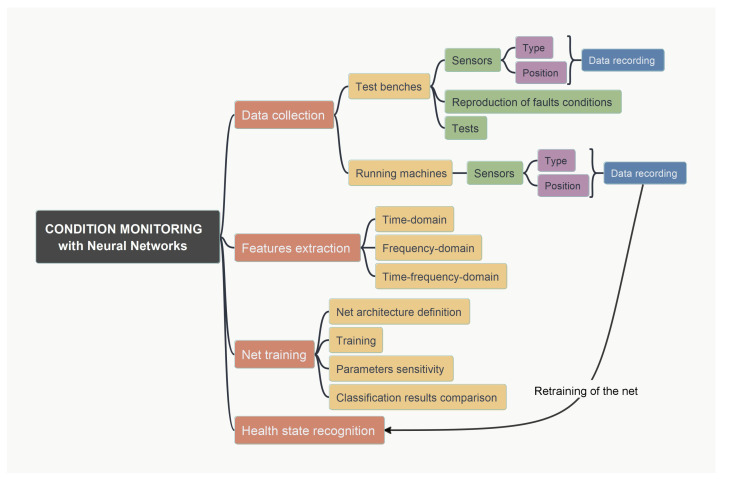
Conceptualization of the main phases of the general approach to the development of condition monitoring based on neural networks of an industrial system.

The main phases of the general approach for the development of condition monitoring based on neural networks of an industrial system are schematically shown in the conceptual map in Figure 1. Since it is a machine learning method, the starting point is data acquisition. Experimental data related to the machine to be monitored can be collected in two main ways: with an experimental approach based on tests performed on a suitably equipped test bench or on the working machine. While the first approach is always possible, the second may only be possible in certain cases. However, conducting tests on a test bench has the advantage that it is possible to collect data on intentionally reproduced fault conditions and thus provide a complete dataset of examples on different operating conditions much faster than field collection. The choice of sensors to be used and their positioning are very important points of the process, because they have a fundamental influence on the results that can be achieved. Once the dataset is available, the next step is to select the features to be extracted and proceed with the relative extraction. This selection also determines the result obtained. The features are the input for the neural network. The network architecture must be selected together with the associated internal parameters and the training options. In research, appropriate sensitivity analyses are often carried out for different parameters. Once the network has been successfully trained, it can be used for field condition monitoring. The continuous collection of data then allows the dataset to be constantly increased, which can enable subsequent retraining of the network to gradually improve the reliability of the classification as the “knowledge” of the machine increases.

**Figure 2:**
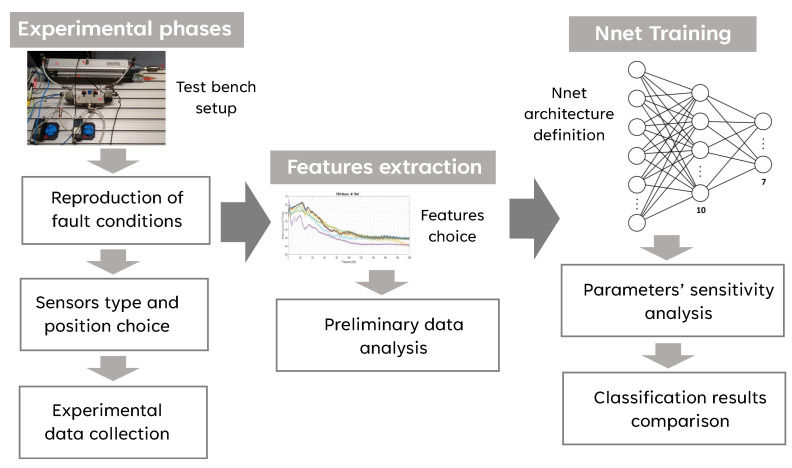
Graphical description of the work-flow of the condition monitoring system development.

The proposed approach is experimental-based. Specific fault operating conditions are reproduced on a test bench. Signals acquired under these operating conditions are previously processed to extract features that are used as input of a feed-forward neural network trained with supervised learning. Part of these data are used for learning, part for verification, and part for validation. The early-stopping method is adopted to prevent the network from becoming too specialized in the learning data, at the expense of its generalization capacity. Figure 2 shows the process followed to develop the condition monitoring system based on supervised classification. The first part of the activity is experimental and involves the replication of operational damage and the acquisition of measurements under these conditions. Of fundamental importance is the choice of sensors to be used and their positioning in the system. One phase of the activity was devoted to the comparison of signals from different sensors and the comparison of signals acquired at different positions. After data collection, features are extracted that allow reducing the dimensionality of the classification problem. The choice of features is fundamental to the quality of the result. After selecting the feature(s) to be used, an initial analysis of the results may be useful to check whether significant differences are found among the operating conditions, or a revision of the feature selection may be necessary. A feed-forward neural network using supervised learning with error backpropagation is used to classify operational states. Once the network architecture is defined, the training and validation of the results can follow. In this phase, several choices have to be made: the structure of the network and its internal parameters, such as the number of levels and the number of neurons for each level, the transfer functions to be used, the partitioning of the examples obtained in the previous phases between learning, validation, and testing.

### 2.2. Experimental Setup

**Figure 3:**
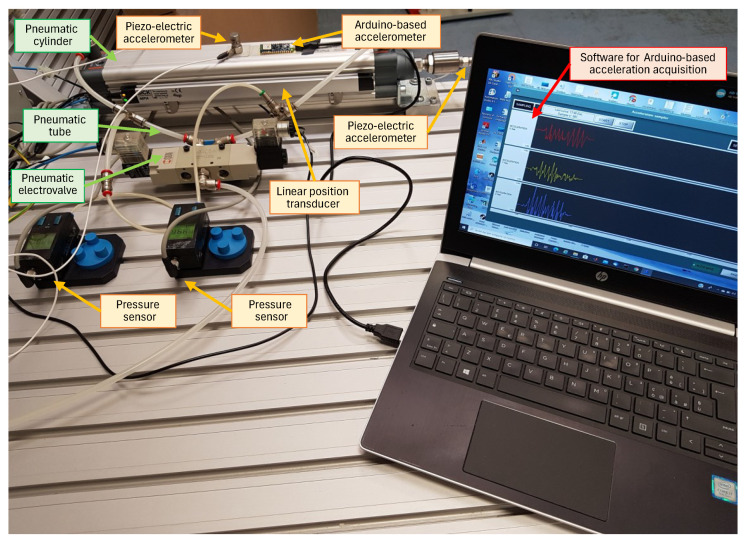
The developed test bench for the acquisition of experimental data under the operating conditions of an electro-pneumatic system.

In an automatic machine, common electro-pneumatic circuits consist of one or more actuators (often double-acting cylinders), electro-valves, a PLC to control the operating cycle, and the pneumatic and electrical connections. In order to experimentally reproduce operational faulty conditions of an electro-pneumatic system, a special test bench was developed (Figure 3), with a double-acting cylinder with a 50 mm rod diameter and a 5/2 bistable electro-pneumatic valve controlled by a 24 V voltage. The reproduced malfunctions (described in detail in Section 2.3) are related to the incorrect mounting of the actuator (a situation that can generate vibrations during movement) or to the presence of air leaks in the pneumatic connections between the components. The system was instrumented with

- two pressure transducers (Festo SDE1 (Esslingen, Germany), with pressure range 0–10 bar), which measure the pressure in the two cylinders’ chambers;
- a linear position transducer (SICK MPA-215THTP0 (Minneapolis, MN, USA), visible in Figure 3) to measure the piston’s position;
- two monoaxial accelerometers (Wilcoxon (Frederick, MD, USA), model 732 A, with frequency range 0.5–25,000 Hz); one axially mounted on the rod, and one radially on the cylinder tube.

The acquisition of the signals from these sensors is performed with National Instruments components (an NI 9201 board for the acquisition of the pressure signals and an NI 9233 for the signals from the accelerometers), with a sampling frequency of 2000 Hz, and with an acquisition program developed in the Labview environment. The command for the pneumatic electro-valve is generated by an NI 9482 card, which takes over the function of the digital output source.

A triaxial accelerometer integrated with an Arduino Nano 33 Bluetooth Low Energy (BLE) Sense Board (Somerville, MA, USA) (visible in Figure 3) is also attached to the cylinder body next to the monoaxial sensor. The Arduino board is directly connected to a PC so that the signal from the accelerometer integrated on the board can be acquired at a very low cost. For this acquisition, the sampling frequency is 500 Hz. An application for interfacing with the Arduino Nano 33 BLE has been developed in the Power-Ki programming environment (developed by XPLAB s.a.s-Research in Automation, in Brescia, Italy, which requires very little development time [40]. Power-Ki is a programming language designed for creating intelligent applications (A.I.), which range from IoT, to monitoring and control systems, to applications for managing production and decision-making processes, as well as web and e-Commerce.

The main technical features of the personal computer used for the acquisition are HP Pavilion Notebook (Palo Alto, CA, USA) with an Intel i7-6700HQ processor (2.6 GHz) (Santa Clara, CA, USA), 8 GB of DDR4 SDRAM (1066.7 MHz), and a Windows 10 Home operating system (Microsoft Corporation, Redmond, WA, USA).

### 2.3. Experiment Description

The electro-pneumatic circuit built on the test bench represents a system widely used in automatic machinery for handling parts. For example, it may represent a typical system for positioning components/parts within an automated assembly island. The malfunctions reproduced on the test bench are

**Faulty attachment of the actuator to the frame.**  Three different situations were considered: the front fixing screws were loosened, the rear fixing screws were loosened, both the front and rear fixing screws were loosened.
**Air leaks in the circuit.**  A hole was made in the connecting pipe between the front chamber and the directional control valve, a hole was made in the connecting pipe between the rear chamber and the directional control valve, and a hole was made in both connecting pipes between the cylinder and the directional control valve. In all cases, the hole has a diameter of 1 mm.

Table 1 lists all operational conditions considered.

The data on the various operating states of the system under investigation were obtained by performing movement cycles of the cylinder under the conditions specified in Table 1 and recording the signals from the sensors mounted on the test bench.

In each test, the pneumatic actuator performed two complete cycles with the pressure set to approximately 4 bar. The physical quantities measured were

- the pressure in the two chambers of the cylinder;
- the displacement of the rod;
- the acceleration of the piston;
- the vibration of the body of the actuator.

**Figure 4:**
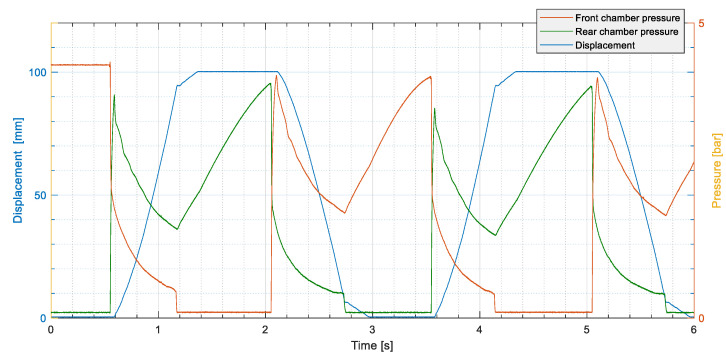
Example of displacement ad pressure signals measured in a test: displacement of the rod in blue; front chamber pressure in orange; rear chamber pressure in green.

**Figure 5:**
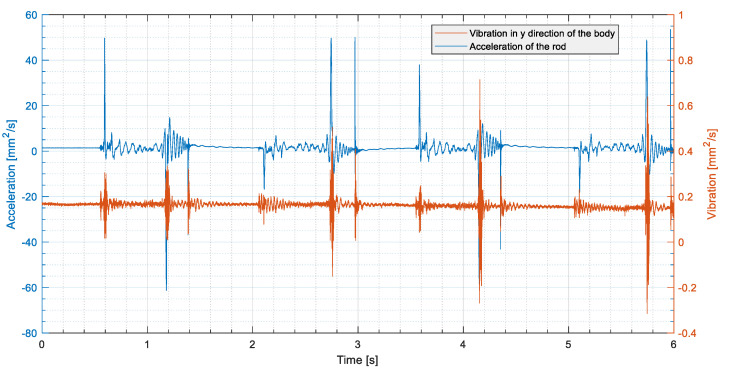
Example of acceleration signals measured in a test: acceleration of the rod in blue, vibration of the cylinder body measured by the piezoelectric accelerometer in orange.

To ensure good repeatability, the tests were repeated 50 times for each operating condition. Figure 4 and Figure 5 show the behaviour of the detected signals in a typical test cycle.

The sensor signals were acquired with two different acquisition systems: one based on National Instruments components and using a software application developed in the Labview programming environment for the pressure sensors and the piezoelectric accelerometers, the other using an Arduino Nano 33 BLE board and a software application developed in the Power Ki programming environment for the accelerometer integrated in the Arduino board. In order to obtain coherent signals, the start of the acquisition of the two different software applications was synchronized.

Table 2 summarizes all experimental tests performed. The dataset based on accelerations measured with piezoelectric accelerometers was labeled M, while the dataset with measurements performed with the Arduino system was labeled A.

### 2.4. Feature Extraction and Dataset Analysis

Theoretically, the measurement of vibrations has the disadvantage that the vibrations at one point of the machine may have been generated by distant sources and propagate to the point at which they were measured. However, this has the advantage that several elements of the machine can be kept under control with a relatively limited number of measurement points.

For condition monitoring of mechanical systems based on supervised classification using vibration measurements, various features can be used as input for classification. The most commonly used features include fast Fourier transform (FFT) and power spectral density (PSD) representations of the signal in the frequency domain [22,41,42,43,44,45].

First, a decision must be made between the use of FFT- or PSD-based features. Preliminary analyses have shown that PSD performs better than FFT in detecting errors in the system under consideration. The PSD has the advantage that it is normalized with respect to the width of the frequency bin, so that it is not influenced by the duration of the sampled signal and thus enables a comparison of different vibrational environments. The duration of the sampling only influences the regularity of the curve, which becomes smoother as the duration of the signal increases. The FFT does not have this advantage, as the frequency resolution is directly proportional to the duration of the signal. The PSD has the further advantage that the power density of the acceleration can be used to obtain the power densities of the velocity and displacement by simply dividing them by $\omega^{2}$ and $\omega^{4}$, respectively. In this way, the same signal also provides information about the energy entering the machine and its structural load. It was therefore decided to use PSD in the further course of the analysis.

The PSD spectra were calculated with the following MATLAB (R2022b) code:

*nfft* = 2(^(nextpow2(length(x)));
  *numWindows* = 8;
  *nWin* = nfft/numWindows;
  *noverlap* = nWin/2;
  *window* = hanning(nWin);
  *(pxx,fx)* = pwelch(x,window,noverlap,nWin,Fs);
  *PdBWx* = 10 * log10(pxx);

where:

*nfft* = points in the x signal;
  *nWin* = samples in the windows;
  *noverlap* = overlapping time samples;
  *Fs* = sampling rate, which was 2000 Hz for the monoaxial accelerometers and about 528 Hz for the Arduino accelerometer.

**Figure 6:**
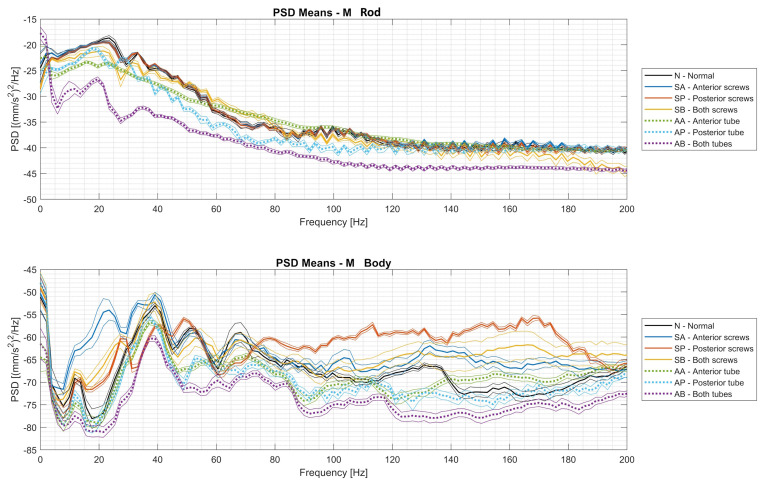
PSD means of the Mr and Mb datasets within the ±1 σ band.

**Figure 7:**
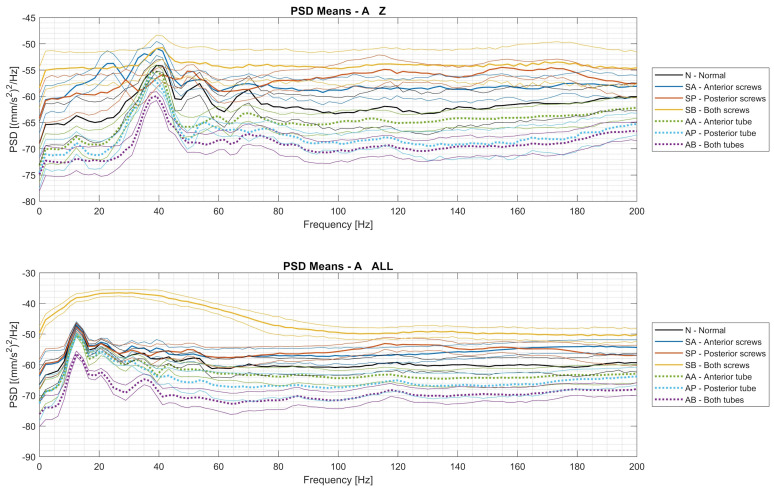
PSD means of the Az and Axyz datasets within the ±1 σ band.

Figure 6 shows the mean PSD curves and the ±1 σ band for the seven operating conditions for the Mr and Mb datasets. Similarly, the average PSD trends and associated ±1 σ bands for the Az and Axyz datasets are shown in Figure 7.

The following considerations can be derived from the analysis of the trends of the PSDs of the Mr and Mb datasets, which are shown in Figure 6:

- the high-frequency components are significant for the vibrations measured on the cylinder body, while they are negligible for the acceleration of the rod;
- poor attachment of the cylinder body to the frame produces amplitudes of the high-frequency components of the Md dataset that are significantly higher than those of the normal case, while there are no large deviations in the amplitudes of the low-frequency components (except around 20 Hz);
- in the operating condition with air leaks, the amplitudes at all frequencies are lower than in the normal state, and the state with air leaks in the direction of both chambers is clearly different at all frequencies.

When analyzing the PSDs of the vibration signals recorded by the Arduino module (Figure 7), the following considerations arise:

- for both the Az and Axyz datasets, the PSD spectra appear to match the energy content of the oscillation, which was maximum when all screws were loosened and minimum when the airflows in both chambers of the actuator were reduced;
- if only the Z component of the oscillation is considered (Az dataset), there is a greater overlap between the signal bands associated with the different conditions;
- when all the screws were loosened (the yellow curves), the PSD of the vibration resulting from the vectorial sum of the X, Y, and Z components was very different from the others, as the actuator oscillated in all three dimensions, causing a more complex phenomenon;
- looking at the overall acceleration, there is a significant peak in the PSD around a frequency of 13 Hz under all operating conditions, with the sole exception of the case in which all screws were loosened;
- a peak at a frequency of just under 40 Hz is present in both the Az and Axyz dataset signals, but it is much more prominent in the z-direction (perpendicular to the body) for all operating conditions.

Comparing the trends of the PSDs between the datasets Mb and Az, which recorded the same vibration, the following considerations arise:

- there is a larger band of signal variability around the average value in Az than in Mb;
- the peaks that characterize the various signals up to 80 Hz are detected in both cases.

### 2.5. Statistical Analysis of the Experimental Data

The collected data were further analyzed by calculating statistical indicators to obtain more useful information about the effects of the sensor positioning, sensor type, and signal components considered in the case of a triaxial sensor.

**Figure 8:**
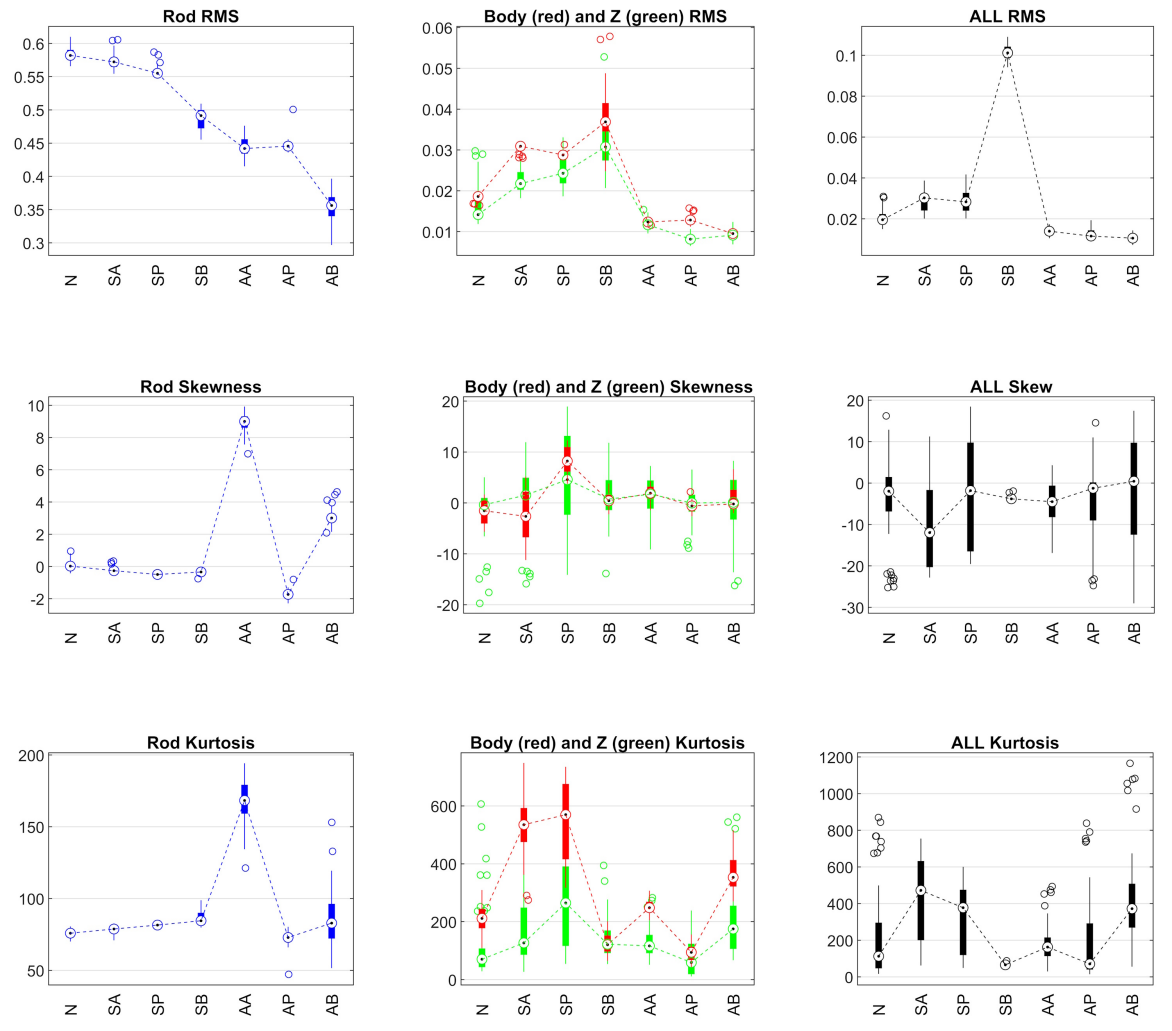
RMS, skewness, and kurtosis statistical indexes of all the acceleration signals. Signals of “Rod” and “Body” are measured by the piezoelectric accelerometer; signals “Z” and “ALL” are measured by the Arduino integrated accelerometer.

It was decided to neglect the peak and crest statistics as they depend on the sampling frequency, which may not capture the true signal peaks if this frequency is low. Therefore, only the RMS value of the signal and the statistics on the shape of the signal distribution (skewness and kurtosis) were considered (Figure 8).

#### 2.5.1. RMS

The signals measured on the rod and on the cylinder body are very different, and consequently the statistical indicators are also very different.

The RMS value is significantly higher at the rod, but decreases with increasing air leakage. On the body, the strongest signals are caused by the loosened bolts (both front and rear), as the vibrations go in the same direction as the accelerometer.

For the Arduino signals, the intensity and trend in the Z direction are similar to the piezoelectric accelerometer measurements on the body. The RMS value of the triaxial Arduino measurement when all bolts were loosened (SB) is much higher than under the other conditions because it combines the vibrations in the three orthogonal directions. In addition, the RMS value of the component in the Z direction allows a better distinction between the different conditions than the RMS value of the signal considering all three components.

#### 2.5.2. Skewness and Kurtosis

The skewness quantifies the asymmetry in the shape of the distribution as the relative size of the tails. Asymmetry manifests as a non-zero value for the standardized third central moment. Figure 8 shows that there is a clear difference between the rod and body signals. The distribution of the rod signal becomes asymmetric when air leaks were present, while this is the case for the body signal when the screws were loose. The skewness of the Arduino signal in the z-direction is similar in shape and intensity to that of the piezoelectric signal on the body, while for the three-dimensional signal, it is not only significantly higher for some defects, but the fluctuation range is also larger.

Kurtosis measures the weight of the two tails of a distribution compared to a normal distribution with the same variance. The distribution is normal if the kurtosis is 3, while the tails are heavier if the kurtosis is greater than 3 and lighter if it is less than 3. In all cases in this study, the kurtosis values are much greater than 3. Again, the difference between the signals measured on the rod and on the body is significant.

Overall, the statistics of the signals of the homologous groups M and A (Mb and Az) show similar trends, albeit with values that depend on their sensitivity.

### 2.6. Adopted AI-Based Classifier

The network for classifying the different faults was created using the Matlab Machine Learning Toolbox. The network receives the features extracted from the signals as input and identifies the fault via the output.

Since the resolution of the PSD was different for the two types of accelerometers due to their different sampling frequency (i.e., sampling frequency of 2000 Hz for the piezoelectric and 500 Hz for the Arduino-integrated accelerometer), the number of inputs for the same maximum frequency was different. Table 3 shows the number of input nodes of the network with the two different accelerometers when the maximum frequency varies. The absolute maximum frequency of the PSD could not be the same as it is half the sampling frequency (1000 Hz for the piezoelectric, about 250 Hz for the Arduino). The number of frequencies was chosen to obtain a reasonable but significant number of cases.

A sensitivity analysis of classification performance was performed, taking into account the effects of the following factors:

- different values of the maximum frequency of the PSD;
- the PSD in dB or not in dB;
- the percentage of data used for testing the net.

**Figure 9:**
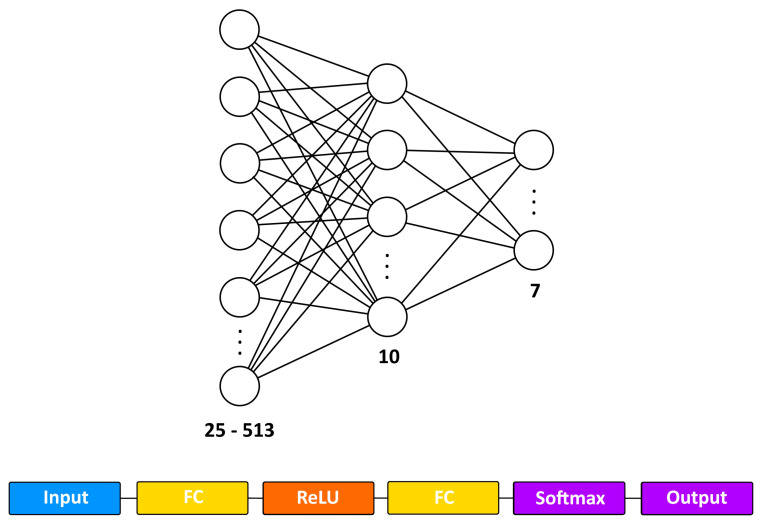
Architecture and configuration of the neural network. Top: network architecture with the input layer (25–513 nodes depending on the PSD), hidden layer (10 nodes), and 7 output nodes (one for the normal state and six for the errors). Bottom: configuration of the fully connected neural network in Matlab with a hidden layer.

The Matlab function used to generate the net was *fitcnet*. It trains a feed-forward, fully connected neural network with a hidden layer corresponding to the structure shown in Figure 9. The neural network receives as input a matrix with the values of the PSD bins (in the rows) for all examples (in the columns). The fully connected input layer (first FC) has a number of nodes corresponding to the PSD bins. The Rectified Linear Unit (ReLU) function is used as the activation function for the nodes of the hidden FC layer, which performs a threshold operation where any value less than zero is set to zero. The Softmax activation function normalizes the output of the hidden FC layer. The last layer is the classification layer, which consists of 7 nodes. This layer uses the probabilities returned by the Softmax activation function for each input to assign the input to one of the mutually exclusive classes.

To summarize, the default Matlab neural network generated by *Fitcnet* has the following characteristics:

1. Input Layer Size: dependent on the features extracted from the acceleration signal;
2. Hidden Layer Size: 5 to 30 nodes;
3. Hidden Layer Activation: ReLU;
4. Output Layer Activation: Softmax;
5. Solver: LBFGS—Broyden–Fletcher–Goldfarb–Shanno quasi-Newton algorithm (LBFGS) as a loss function minimization technique, where the software minimizes the cross-entropy loss.

*Fitcnet* applies the *Softmax* function (Equation (1)) to the last fully connected layer to obtain the predicted classification results (or posterior probabilities).

(1) $$f\left(x_{i}\right)=\frac{exp(x_{i})}{\sum_{j=1}^{K}exp\left(x_{i}\right)}$$

where $x_{i}$ are the inputs and *K* is the number of classes in the response variable (type of fault).

The function *Loss* returns the classification loss *L*, which measures the prediction inaccuracy of the classification model: lower losses indicate better prediction models. *Mincost* is the standard loss function, which is only suitable if the classification results are posterior probabilities (as in this case). It calculates the weighted average of the minimum expected misclassification cost losses, according to Equation (2).

(2) $$L=\sum_{j=1}^{n}w_{j}c_{j}$$

where *j* are the observations, $w_{j}$ are the normalized observation weights, and $c_{j}$ are the costs of producing the predictions.

## 3. Results

The accuracy of the classification used in the training process of the neural network is calculated as (1 − *L*), where *L* is calculated by keeping the default setting of the Matlab function *LossFun*, i.e., *Mincost* (Equation (2)).

Table 4 shows the total number of signals and their distribution between training and test examples for the dataset M (measurements with piezoelectric accelerometers) and for the dataset A (measurements with integrated Arduino accelerometer). From the set of training examples, a validation subset is automatically extracted from the functions implemented in Matlab, which is used for the application of the early-stopping method to avoid excessive specialization on the training examples and thus a lower generalization capacity.

### 3.1. Classification Based on PSD of Acceleration Signals

In the classification with ANN with PSD of acceleration data as input, the influence of the following factors was investigated:

- the maximum frequency of the PSD;
- the number of neurons in the hidden layer;
- the percentage of data used for training the nets.

To analyze the effect of the number of neurons in the hidden layer, a range of 5 to 30 neurons with intervals of 5 neurons (5–10–15–20–25–30 neurons) was defined.

**Figure 10:**
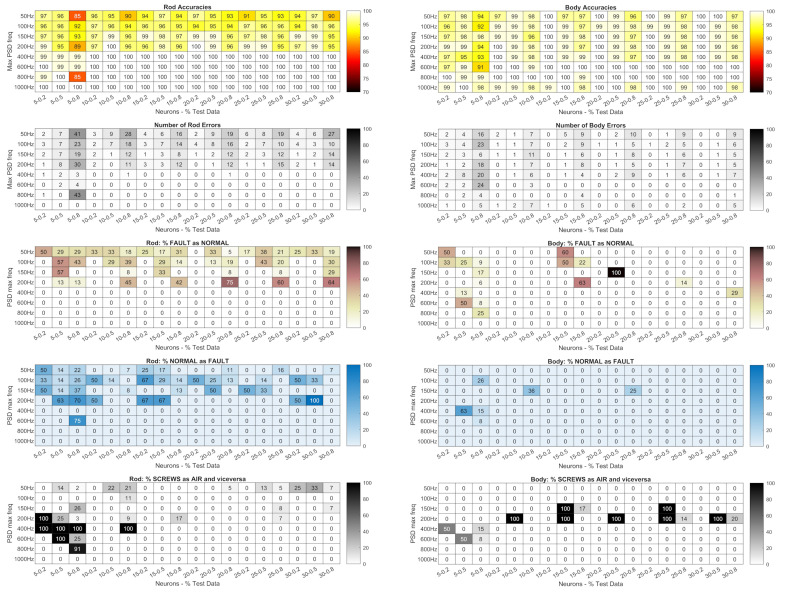
Piezoelectric accelerometers: accuracy of the networks trained with the PSD in dB; total number of errors (heatmaps with gray shading); percentage of faults not detected (heatmaps with brown shading); percentage of normal conditions identified as faults (heatmaps with blue shading); percentage of classifications of the wrong fault type (screws as air or vice versa, the heatmaps at the bottom with gray shading). Lighter colour gradations mean better results.

**Figure 11:**
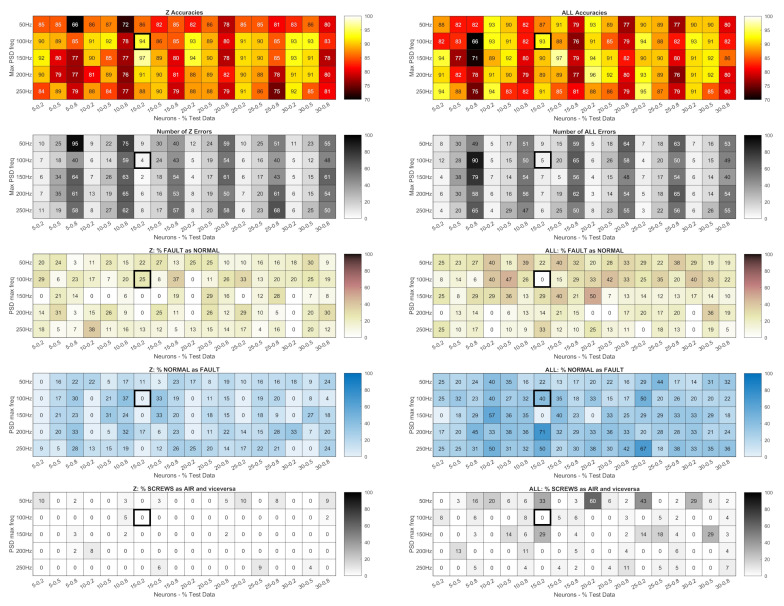
Arduino accelerometer: accuracy of the networks trained with the PSD in dB; total number of errors (heatmaps with gray shading); percentage of faults not detected (heatmaps with brown shading); percentage of normal states detected as faults (heatmaps with blue shading); percentage of classifications of the wrong type of fault (screws as air or vice versa, the heatmaps at the bottom with gray shading). Lighter colour gradations mean better results.

The heatmaps in Figure 10 and Figure 11 show the accuracy of the networks trained with the PSD in dB of the signals measured by

- the piezoelectric accelerometers (Figure 10);
- the Arduino accelerometer (Figure 11).

The figures also show the number of errors (heatmaps with gray shading); percentage of faults not detected (heatmaps with brown shading); percentage of normal conditions identified as faults (heatmaps with blue shading); percentage of the classification of the wrong type of fault (screws as air or vice versa, the heatmaps at the bottom with gray shading). Lighter colour gradations mean better results. Errors in the classification of the same type of fault (screws or air) are not shown as they are the complement to 100% of the sum of all the above errors.

Each result is related to

- the maximum frequency of the PSD (the row parameter);
- the number of neurons in the hidden layer (column parameters on the left);
- the percentage of data used in the tests compared to the total data (column parameters on the right, expressed as decimals).

A preliminary analysis showed that with the piezoelectric accelerometers, the way the PSD is represented (in dB or not) has no influence, as the difference in net accuracies between the representations is only 2% for measurements on the body and 4% for measurements on the rod. For the Arduino signals, a larger spread of differences can be observed (with variations of up to 15%) and the average values were more skewed toward the dB representation. Therefore, all of the following analysis is based on PSDs in dB.

### 3.2. Classification with the Statistics of the Signals

In the classification with ANN based on the statistics of acceleration data, the influence of the following factors was studied:

- the number of neurons in the hidden layer;
- the number of statistics;
- the percentage of data used for training the nets.

Different combinations of statistics could be used as input for the ANN, but of all possible ones, only those in Table 5 were considered.

**Figure 12:**
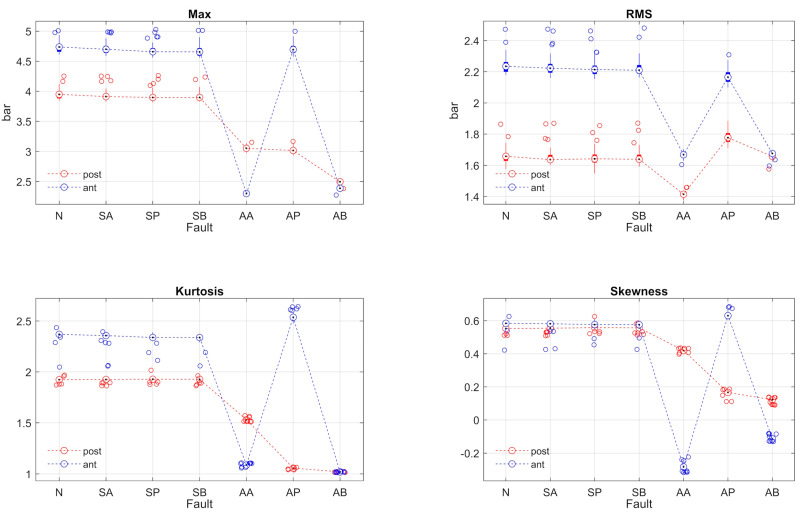
Peak, RMS, skewness, and kurtosis of the pressure signals.

The group of 15 statistics contains them all. A selection process was then carried out based on the original group. To obtain the group of 13 statistics, the peak and crest values of the vibrations have been removed, as their value is influenced by the sampling frequency. This problem does not occur with pressure signals. To obtain the group of 9 statistics, crest and kurtosis of the pressure signals were removed from the group of 13, as they show the same trend as peak and skewness (see Figure 8 and Figure 12). Kurtosis and skewness of the vibrations and of the pressures are removed from the group of nine, leading to the group of five statistics: the reason for this is to evaluate whether the RMS values associated with the energy of the phenomenon allow a correct detection of the faults. However, the peak signal has been kept for the pressures as it allows a better discrimination of the type of faults, which has a lower energy content than that of loosening bolts. Finally, the group of three statistics contains only the RMS values.

**Figure 13:**
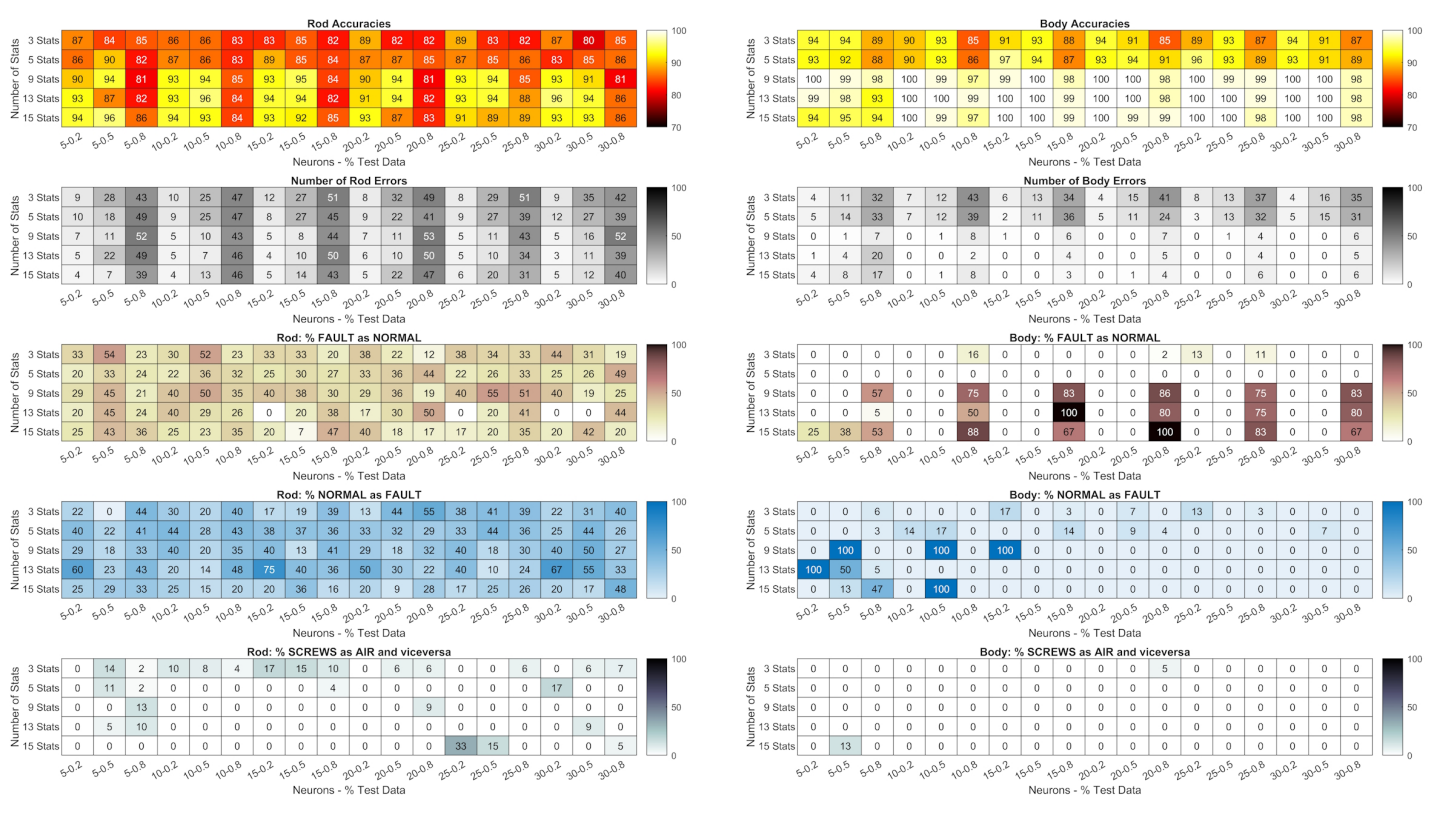
Piezoelectric accelerometers: accuracy of the networks trained with statistics; total number of errors (heatmaps with gray shading); percentage of errors not detected (heatmaps with brown shading); percentage of normal conditions detected as errors (heatmaps with blue shading); percentage of classifications of the wrong error type (screws as air or vice versa, the heatmaps at the bottom with gray shading). Lighter colour gradations mean better results.

**Figure 14:**
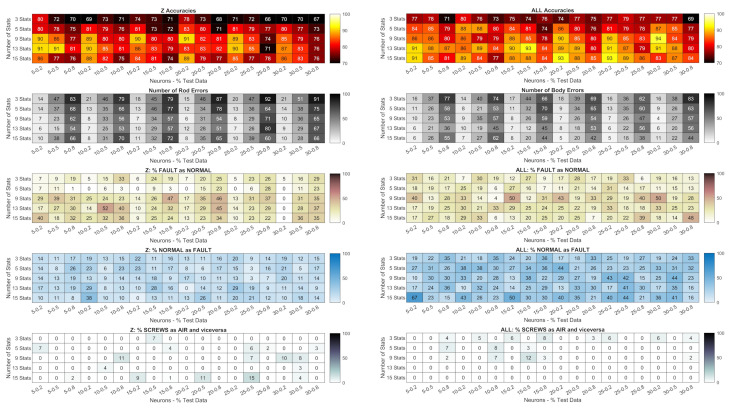
Arduino accelerometer: accuracy of networks trained with statistics; total number of errors (heatmaps with gray shading); percentage of errors not detected (heatmaps with brown shading); percentage of normal conditions identified as errors (heatmaps with blue shading); percentage of classifications of the wrong fault type (screws as air or vice versa, the heatmaps at the bottom with gray shading). Lighter colour gradations mean better results.

Figure 13 and Figure 14 show the results of the classification with the combinations of statistics (rows) in Table 5, for different numbers of neurons and test data fractions (columns). Lighter colour gradations mean better results.

## 4. Discussion

### 4.1. Classification Based on PSD of Acceleration Signals

The heatmaps in Figure 11 show that networks built with signals measured on the body offer higher accuracy on average. However, in the case of rod measurements, the networks built with a PSD of 400 Hz or more and 10 or more neurons provide an accuracy of 100%. For body measurements under the same conditions, the accuracy is sometimes below 100%, but it never happens that normally, they are considered as normal conditions. This also applies to frequencies of 150 Hz and 200 Hz with 20% test data.

Classification with the Arduino signals is much less accurate (Figure 11). However, the networks trained with PSDs of the Z signals with 150 Hz and 80% of the training examples performed extremely well and avoided the most severe errors, i.e., normal states considered as faults and especially not-recognized faults.

However, to properly evaluate the accuracy of a network, the type of error must also be considered. In general, the most serious errors are those where a fault is not detected, as they could affect either the integrity of the machine or its efficiency and precision.

In the case of faults being detected when none are present, only efficiency is impaired because the machine is stopped for inspections even though there is no need. Less serious errors are the identification of the wrong type of fault, i.e., a loosening of the screws classified as an air leak or vice versa: in this case, the inspection of the machine is prolonged and therefore the time it remains unproductive. Finally, the least serious errors are those where the nature of the fault has been correctly identified, but not its position. As an example, we can analyze the case of Arduino classifications for networks with 15 nodes in the hidden layer trained with PSDs of Z and ALL signals with 100 Hz and 80% (highlighted by black rectangles in Figure 11). The accuracies are good (94% for the Z signals and 93% for the ALL signals), and the total errors are four and five, respectively. However, the network with the highest accuracy did not detect any fault in one case, while the other network reported two faults even though the condition was normal.

Consequently, even a network with an accuracy close to 100% might be unsatisfactory, if it reduces the efficiency of the machine by reporting non-existent faults, or compromises its integrity by not reporting existing faults. Therefore, it is always necessary to analyze the nature of the error to be sure that the network is effective because accuracy alone is not enough. If this is not possible or too costly, an accuracy function must be defined that takes into account the severity of the error.

### 4.2. Classification with the Statistics of the Vibrational and Pressure Signals

As far as monoaxial accelerometers are concerned, classification by statistics can be extremely accurate. Body measurement with many combinations of statistics and the number of neurons can achieve 100% accuracy. In general, at least 9 statistics and 10 neurons are needed to achieve the best results.

Also noteworthy is the fact that the body measurements never misclassified faults as normal states, which is the worst possible outcome as it prevents potentially harmful situations from being recognized. The same applies to the reverse error, i.e., normal states interpreted as faults. In this case, however, a detection error would only lead to a reduction in the efficiency of the machine, as it would be subjected to unnecessary maintenance. On the contrary, the case with the measurement on the rod is not so precise, and no combination achieves an accuracy of 100%. It is also difficult to find a combination of statistics and data that is preferable to others.

The Arduino delivers significantly worse results than the monoaxial accelerometers, as the accuracy is worse on average. However, the results show how misleading it may be to rely on accuracy alone to judge the precision of a network. The combined signals of the three axes (ALL) seem to be preferable as they generally provide higher accuracy. However, networks with 30 nodes trained with 80% of orthogonal signals (Z) never make the mistake of classifying faults as normal states, showing that they are more reliable than all others in this respect.

### 4.3. Errors

Since detection errors can have very different consequences, some of which are tolerable while others are potentially harmful, the nature of these errors must be taken into account when evaluating the accuracy of a network.

**Figure 15:**
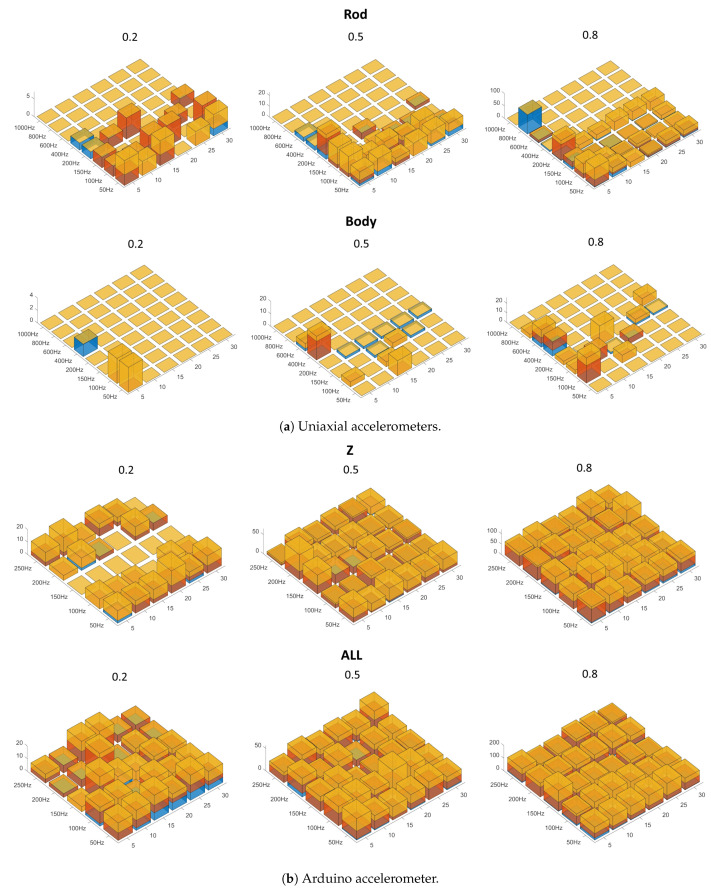
Error value with PSDs as input data. Yellow = unrecognized faults times 4; red = normal conditions mistaken for faults times 3; blue = identification of the wrong type of fault (screws as air or vice versa) times 2. The PSD spectra are on the left axes; the number of neurons in the hidden layer are on the right axes; the error values are on the vertical axes; the percentages of data used for testing the nets are at the top of each graph.

**Figure 16:**
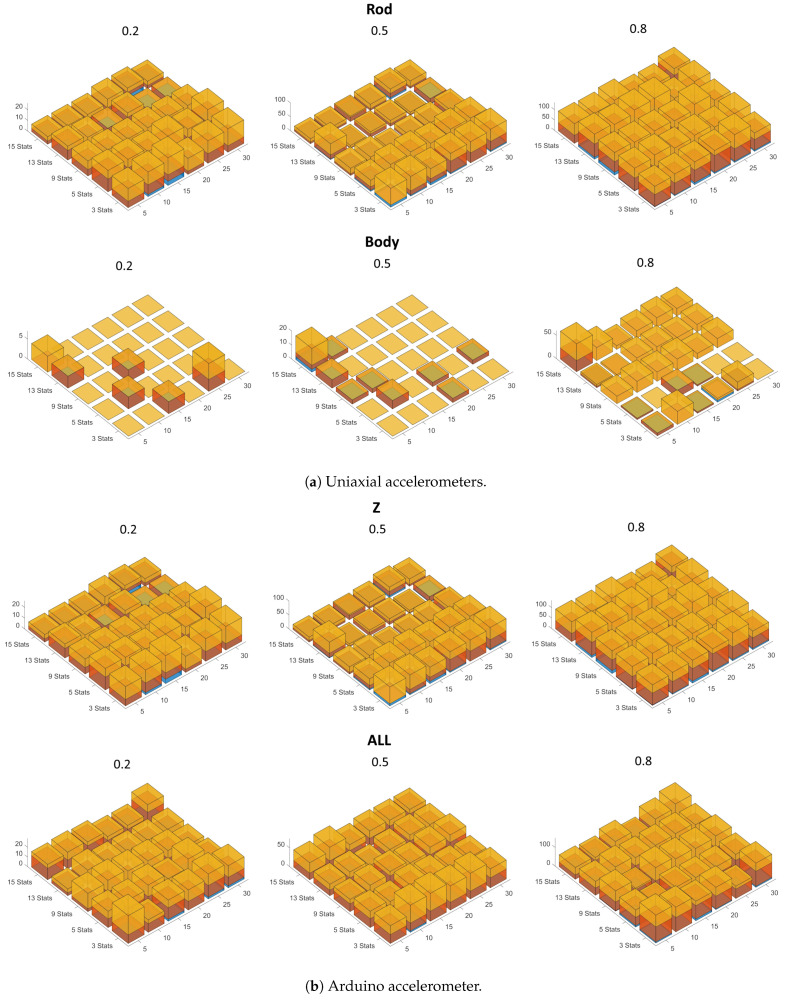
Error value with statistics as input data. Yellow = unrecognized faults times 4; red = normal conditions mistaken for faults times 3; blue = identification of the wrong type of fault (screws as air or vice versa) times 2. The numbers of statistics are on the left axes; the number of neurons in the hidden layer are on the right axes; the error values are on the vertical axes; the percentages of data used for testing the nets are at the top of each graph.

One method is to assign weights to errors to consider their severity. In this work, a simple and abstract criterion was defined. The total number of occurrences of a particular type of error was multiplied by a weighting to obtain a value that correlates with the frequency of the error itself and its severity. Then, all these values were added up and compared graphically in stacked bar charts, where the higher the bar, the greater the total error, while the colors indicate how much each error type contributes to this error (*error value* in Figure 15 and Figure 16).

Despite its simplicity and abstractness, the method is useful to better understand the influence of the amplitude of the PSD spectra, the number and type of statistics, and finally the number of neurons on the performance of the network.

For the sake of simplicity, only the three types of error that should theoretically be the most serious were considered. The following is in decreasing order of severity: unrecognized faults; normal conditions mistaken for faults; the identification of the wrong type of fault (screws as air or vice versa). The corresponding weights (or multipliers) were, respectively, 4; 3; 2. The colors are, respectively, yellow; red; blue.

#### 4.3.1. PSD

Figure 15 shows the *error value* when the PSDs were used as input to the networks.

The monoaxial acceleration sensors deliver much better results than the Arduino: the error value (sum of the number of occurrences multiplied by the respective weighting) is significantly lower, although Z signals with a PSD of 150 Hz never show serious errors, regardless of the number of nodes, when trained with 80% of the signals.

With monoaxial accelerometers, the measurement on the body is better than on the rod and provides good overall accuracy, even when training with only 20% of the signals. In general, it is better to use networks that have at least 10 nodes.

In the case of the Arduino, the Z measurements provide better results than the ALL measurements. In addition to the previously reported case of no serious errors with a PSD with 150 Hz, the networks with 20 neurons also perform well when trained with the largest number of signals and a PSD spectrum of 100 Hz or 200 Hz.

#### 4.3.2. Statistics

Figure 16 shows the error value when the statistics were used as input for the networks. For networks trained with monoaxial body measurements, far less severe errors occur than for all other networks. Even networks trained on up to 50% of the data often do not make the most severe error (unrecognized faults, in yellow).

In general, the difference in quality between all the networks is not as pronounced as when training with PSDs, so networks trained with the statistics of the Arduino signals (and pressures) give comparable results to those trained with the signals measured on the rod, although with lower accuracy (Figure 13 and Figure 14).

## 5. Conclusions

Neural networks are an effective tool for monitoring the state of a device or machine. However, the results they provide depend heavily on the dataset used to create them and the choices for their parameters.

The data must be recorded and processed reliably and accurately to eliminate noise and errors and extract the features that best capture the state of the device. The network must then be created and trained accordingly. This study investigated how some elements of this process affect the NN-based classification of the operating conditions of a pneumatic system.

A comparison was developed between two different types of accelerometers and their positions on the pneumatic system. In the case of piezoelectric accelerometers, the measurements on the body proved to be more accurate overall than those on the rod. With the Arduino-based solution, there was no significant difference between the results of the signals in one direction (Z) and those in all three directions together (ALL).

Although the accuracy of the classification based on the data acquired with the accelerometer integrated in Arduino is lower than that of the data acquired with the piezo accelerometers, it is still high and can be acceptable for many applications. This result is remarkable because the total cost of both the sensor and the acquisition system that characterizes this solution is significantly lower than the piezoelectric sensor solution. Its implications in terms of the feasibility of using low-cost sensors integrated into an Arduino board for reliable vibration-based condition monitoring could have an important impact on the adoption of machine condition monitoring strategies in industrial environments, where pneumatics is very widespread, favoring a wider diffusion of condition monitoring techniques based on vibration signals even for not particularly expensive machines.

In general, the network has to be trained with an appropriate number of signals, which does not necessarily have to be the highest possible, as shown by the results of piezoelectric accelerometers when the PSDs have a spectrum of at least 400 Hz.

The number of neurons is a parameter that has little influence on the accuracy of the network.

For the PSD components to be considered as input to the neural network, better results were generally obtained when the width of the spectrum increased; but for the body dataset, the accuracy was 100% even with a spectrum of only 50 Hz. So, there seems to be an optimal combination that saves both the amount of training data and the calculation of the spectrum. The way the PSD is presented is essentially irrelevant.

When comparing PSDs and statistics, the PSDs for uniaxial accelerometers are preferable as they provide better results. However, the statistics also provided excellent results: for the body, they provided an accuracy of 100% when the statistics were at least nine and the training took place with at least 50% of the signals. The PSDs gave a surprising result for the Arduino Z signals when they had a spectrum of 150 Hz and the training took place with 80% of the signals, as they did not make serious errors regardless of the number of neurons (Figure 15b). When the percentage of training data drops to 20% or ALL signals are considered, the difference becomes less clear, and in the case of the networks trained with the minimum amount of ALL signals, the statistics gave significantly better results.

Finally, the investigation revealed that the type of error also needs to be considered for accuracy, and an approach based on a specific error index is presented.

Having a reliable dataset is very important for machine learning techniques like artificial neural networks. For this reason, the most critical point of the presented approach is the availability of examples related to healthy conditions and malfunctions or fault conditions. This means that they must be obtained from tests on test benches or collected over time from measurements on already functioning machines. Such a solution could therefore mean costly preliminary work if the machines are complex or if the time between design and commissioning is so short that preliminary tests are not possible. This means that they have to be obtained from tests on test benches or collected over time from measurements on machines that are already working. Such a solution could therefore mean costly preliminary work if the machines are complex or if the time between construction and commissioning is so short that preliminary tests are not possible. This critical issue could be eliminated by using synthetic example generation techniques as presented in the literature by many authors [4,5,39,46]. Future activities will focus on the development of models and methods that enable the generation of reliable synthetic examples for the pneumatic system under consideration.

Furthermore, as the piston performs a reciprocating motion, the spectral content changes during the actuation cycle, so it could be tested whether the spectrograms provide useful information for fault detection.

Finally, it would be useful to consider other causes of malfunctioning, such as damage to the seals.

## Figures and Tables

**Table 1:** Considered operational faults and corresponding code.

Condition Code	Short Condition Code	Operating Condition
Normal	N	No faults
Screws-Ant	SA	Loosened anterior screws
Screws-Post	SP	Loosened posterior screws
Screws-Both	SB	Loosened screws
Air-Ant	AA	Air leak in the connection with the anterior chamber
Air-Post	AP	Air leak in the connection with the posterior chamber
Air-Both	AB	Air leak in the connection with both the chambers

**Table 2:** Synthetic overview of the experimental datasets.

Code	Accelerometer	Position	Signal	Faults	Repetitions	Total Acquisitions
Mr	Piezoelectric Monoaxial	Rod	Rod axis	7	50	350
Mb	Piezoelectric Monoaxial	Body	Rod axis	7	50	350
Az	Arduino Tri-axial	Body	Axis perpendicular to the frame	7	50	350
Axyz	Arduino Tri-axial	Body	X, Y, Z axis	7	50	350

**Table 3:** Number of net input nodes.

Max PSD Frequency	Piezoelectric	Arduino
50	-	25
100	52	49
150	-	73
200	103	97
250	-	122
400	205	-
600	308	-
800	410	-
1000	513	-

**Table 4:** Training and testing signals. The “Data Size” column indicates the total number of signals for each dataset. Dataset M: measurements with piezoelectric accelerometers. Dataset A: measurements with Arduino-integrated accelerometer.

Dataset	Data	Percentage of Test Data						
	Size	20%	30%	40%	50%	60%	70%	80%
M	350	280/70	245/105	210/140	175/175	140/210	105/245	70/280
A	336	269/67	235/101	202/134	168/168	134/202	101/235	67/269

**Table 5:** Groups of statistics.

Stats	Vibration	Anterior Pressure	Posterior Pressure
3	RMS	RMS	RMS
5	RMS	RMS, peak	RMS, peak
9	RMS, kurt, skew	peak, RMS, skew	peak, RMS, skew
13	RMS, kurt, skew	peak, RMS, crest, kurt, skew	peak, RMS, crest, kurt, skew
15	peak, RMS, crest, kurt, skew	peak, RMS, crest, kurt, skew	peak, RMS, crest, kurt, skew

## Data Availability

The data presented in this study are available on request from the corresponding author.
